# Dietary Organic Zinc Supplementation Modifies the Oxidative Genes via RORγ and Epigenetic Regulations in the Ileum of Broiler Chickens Exposed to High-Temperature Stress

**DOI:** 10.3390/antiox13091079

**Published:** 2024-09-04

**Authors:** Saber Y. Adam, Madesh Muniyappan, Hao Huang, Wael Ennab, Hao-Yu Liu, Abdelkareem A. Ahmed, Ming-an Sun, Tadelle Dessie, In Ho Kim, Yun Hu, Xugang Luo, Demin Cai

**Affiliations:** 1 Laboratory of Animal Physiology and Molecular Nutrition, Jiangsu Key Laboratory of Animal Genetic Breeding and Molecular Design, College of Animal Science and Technology, Yangzhou University, Yangzhou 225009, China; mh23108@stu.yzu.edu.cn (S.Y.A.); 008664@yzu.edu.cn (M.M.); 221902107@stu.yzu.edu.cn (H.H.); 008568@yzu.edu.cn (W.E.); 007725@yzu.edu.cn (H.-Y.L.); huyun@yzu.edu.cn (Y.H.); 2 International Joint Research Laboratory in Universities of Jiangsu Province of China for Domestic Animal Germplasm Resources and Genetic Improvement, Yangzhou 225009, China; 3 Department of Veterinary Biomedical Sciences, Botswana University of Agriculture and Agriculture and Natural Resources, Gaborone P.O. Box 100, Botswana; aabdallah@buan.ac.bw; 4 Biomeidcal Research Institute, Darfur University College, Nyala P.O. Box 160, South Darfur State, Sudan; 5 Department of Physiology and Biochemistry, Faculty of Veterinary Science, University of Nyala, Nyala P.O. Box 155, South Darfur State, Sudan; 6 Institute of Comparative Medicine, College of Veterinary Medicine, Yangzhou University, Yangzhou 225009, China; mingansun@yzu.edu.cn; 7 International Livestock Research Institute, Addis Ababa 5689, Ethiopia; t.dessie@cgiar.org; 8 Department of Animal Resource and Science, Dankook University, Cheonan-si 31116, Choongnam, Republic of Korea; inhokim@dankook.ac.kr

**Keywords:** zinc, oxidative stress, ileum, RORγ, epigenetics

## Abstract

Heat stress (HS) is a significant concern in broiler chickens, which is vital for global meat supply in the dynamic field of poultry farming. The impact of heat stress on the ileum and its influence on the redox homeostatic genes in chickens remains unclear. We hypothesized that adding zinc to the feed of heat-stressed broilers would improve their resilience to heat stress. However, this study aimed to explore the effects of organic zinc supplementation under HS conditions on broiler chickens’ intestinal histology and regulation of HS index genes. In this study, 512 Xueshan chickens were divided into four groups: vehicle, HS, 60 mg/kg zinc, and HS + 60 mg/kg zinc groups. Findings revealed that zinc supply positively increased the VH and VH: CD in the ileum of the broilers compared to the HS group, while CD and VW decreased in Zn and HS+Zn supplemented broilers. Zn administration significantly increased superoxide dismutase (*SOD*), catalase (*CAT*), glutathione (*GSH*), and decreased the enzymatic activities of reactive oxygen species (*ROS*) and malondialdehyde (MDA) compared to the HS group. In addition, Zn administration significantly increased relative ATP, complex I, III, and V enzyme activity compared to the HS group. Furthermore, the expression of acyl-CoA synthetase long-chain family member 4 (*ACSL4*), lactate transporter 3 (*LPCAT3*), peroxiredoxin (*PRX*), and transferrin receptor (*TFRC*) in the protein levels was extremely downregulated in HS+Zn compared to the HS group. Zn supply significantly decreased the enrichment of *RORγ*, *P300*, and *SRC1* at target loci of *ACSL4*, *LPCAT3*, and *PRX* compared to the HS group. The occupancies of histone active marks *H3K9ac*, *H3K18ac*, *H3K27ac, H3K4me1*, and *H3K18bhb* at the locus of *ACSL4* and *LPCAT3* were significantly decreased in HS+Zn compared to the HS group. Moreover, *H3K9la* and *H3K18la* at the locus of *ACSL4* and *LPCAT3* were significantly decreased in HS+Zn compared to the HS group. This study emphasizes that organic Zn is a potential strategy for modulating the oxidative genes *ACSL4*, *LPCAT3*, *PRX*, and *TFRC* in the ileum of chickens via nuclear receptor *RORγ* regulation and histone modifications.

## 1. Introduction

The global livestock trade is facing main challenges due to heat stress, which greatly affects both animal health and productivity [1]. These impacts lead to increased livestock morbidity and mortality and a significant decline in growth performance, which may result in substantial financial losses [2]. As global temperatures rise, this issue is of great concern, particularly in the major tropical and subtropical animal-producing regions [3]. Chicken is among the animals most susceptible to heat stress [4]. The result is a decline in meat quality and egg production, a compromised immune system, and impaired growth performance [5]. Studies have shown that heat stress leads to alterations in meat color parameters, changes in muscle structure, and increased drip and cooking losses, affecting meat quality [6,7]. Reduced feed intake during heat stress explains the decline in productivity, with intestinal hyperpermeability playing a vital role in nutrient partitioning and immune activation [8]. Moreover, heat stress compromises the immune system by upregulating interleukin expression and reducing antioxidant capacity, mainly in normal-sized chickens compared to dwarf chickens [6]. The body’s homeostasis between antioxidant and antioxidative systems is upset by oxidative stress induced by HS, which is the primary source of these effects [9]. It is well known that HS conditions usually generate reactive oxygen species (*ROS*), which can lead to oxidative damage and the reduction in oxidative capability [10]. Although stress prevention in poultry farms is almost impossible, several techniques are effectively used to reduce the negative effects of heat stress on chickens [11]. Cheng et al. [12] reported that oxidative stress can cause damage to cellular molecules such as DNA, proteins, and lipids, as well as cell dysfunction and tissue injury. It has previously been considered one of the most vexing problems in the contemporary poultry business [13]. HS causes various physiological changes in chickens, including oxidative damage, acid-base imbalance, and suppressed immune function. As a result of heat stress, birds attempt to dissipate heat by evaporative cooling, which raises rectal temperatures and respiration rates [14]. The heat stress interrupts electrolyte balance, which can impair panting as the body seeks to maintain homeostasis [15]. Heat Shock Proteins (HSPs): In response to heat stress, especially *HSP70*, are increased, supporting cellular protection and function and, indirectly, respiratory efficiency during panting [16]. These changes result in reduced feed intake, poor feed efficiency, reduced body weight, lower-quality meat, increased disease incidence, and increased mortality [17]. The demand for poultry increases along with the worldwide population since it is the most widely recognized and popular animal protein. To accommodate this need, poultry genetics has improved significantly over the past several decades [18]. These improved strains are more sensitive to high ambient temperatures and exhibit an increased rate of metabolic and productive capacity [19]. HS affects the uptake of important non-enzymatic antioxidants vitamins E and C and selenium [20] and reduces the activity of key antioxidant enzymes such as glutathione peroxidase (*GSH-Px*) and superoxide dismutase (*SOD*) [3]. Increasing the antioxidant capacity of chickens can reduce the negative impacts of heat stress in response to these problems, establishing the foundation for a more resilient and sustainable poultry sector in climate change [21]. Several approaches, including the administration of vitamins, minerals, antioxidants, and plant extracts, have been used to reduce the detrimental impact of ambient heat stress on chickens by inducing oxidative stress [11]. The development of zinc (Zn) with antioxidant activities has improved chicken heat stress [4,22].

Micromineral feed enrichment is one of the most common and essential strategies in poultry production as an important nutritional approach [23,24]. For the minerals in the diet to be used by the tissues, they must pass through the GI tract’s epithelial cells before reaching the blood [25]. Minerals can be absorbed from any portion of the GI tract. Unfortunately, interactions between minerals within the diet can occur within the digestive tract, particularly for essential trace elements like copper, zinc, iron, and calcium that impair their absorption. For example, calcium can increase iron bioavailability when appropriately fractionated with proteins by minimizing the formation of insoluble complexes that hinder absorption [26]. Vitamin D status is crucial for calcium and phosphate absorption, with its deficiency leading to reduced absorption efficiency [27]. In addition, at various ratios, a 50 μM concentration of zinc reduced cellular copper uptake, demonstrating competitive inhibition [28]. The complexity of mineral absorption is emphasized by these interactions, but they also highlight the necessity of a balanced diet to maximize nutrient bioavailability. Zinc is one of the most abundant trace elements after iron and is essential for all living organisms [29]. Farmers in the summer months, intensively used Zn as a dietary approach to alleviate the negative impacts of heat stress on the animals and to improve productivity [30]. According to some studies, organic zinc has a higher bioavailability than inorganic zinc [31]. Zn-like chemical compounds can reduce oxidative stress in chickens [32]. Zinc improves chicken immunity, increases feed conversion rate, promotes healthy growth and development, and prevents diseases [4,33]. Zn is an essential micronutrient that affects many biological processes in birds such as the immune system, hormone synthesis, protein and DNA synthesis, digestion of carbohydrates, fats, and proteins, and antioxidants [34]. Zinc additionally increases the antioxidant capacity of chickens by influencing gene expression through modifications to DNA and chromatin structure, as well as by enhancing the activity of zinc metalloenzymes and copper-zinc superoxide dismutase [35].

Peroxisome proliferator-activated receptor alpha (*PPARα*) is a ligand-activated transcription factor that belongs to the superfamily of nuclear receptors [36]. The *PPARA/NR1C1* gene encodes *PPAR α*, a transcription factor that senses nutrients and is involved in the transfer of fatty acids and their hepatic β-oxidation. Because of this, the liver, heart, skeletal muscle, and kidney contain high levels of *PPAR α* protein. *PPARs* typically enlist the help of *RXR* partner proteins to bind the cognate *PPAR* response elements (*PPRE*) of their target gene promoters [37]. Hepatocyte-specific *PPARα* mutant animals were shown to accumulate lipids in the liver and gut rapidly [3,38]. Along with the related *RORα* and *RORβ, RAR*-related orphan receptor gamma (*RORγ*) is a subfamily of the nuclear receptor superfamily of transcription factors that includes therapeutic targets for autoimmune and metabolic disorders [39]. Both *RORα* and *RORγ* have significant functions in regulating the circadian rhythmic expression of lipid and glucose metabolism genes in the liver and intestine [40]. *RORγ* is one of the rare nuclear receptors with an unsolved structure, mainly because receptors cannot have multiple ligands. However, modeling predicts the presence of a well-designed hydrophobic pocket, suggesting that physiological ligands can modulate *RORγ* [41]. Antioxidant response element-related proteins such as *GSH*, *SOD*, and *CAT* are increased during stimulation of the *RORγ* pathway [42].

Epigenetic regulation modifies the expression of genes or their rate of expression by DNA methylation, histone modifications, and other regulatory pathways [43]. DNA methylation generally takes place in the promoter region, where it prevents protein binding and, as a result, suppresses the transcription of genes [44]. Apart from DNA methylation, histone modifications can also alter the transcriptional regulation of specific genes via affecting chromosomal domains [45]. Heat stress causes a decrease in H3 methylation as well as an increase in H2B methylation. Rat astrocyte and cortical neuronal cultures have identified the presence of several distinct methylated amino acid residues of H3, particularly H3 at lysine 4 (*H3K4*) and H3 at lysine 9 (*H3K9*). *H3K4* methylation is tied with the activation of gene transcription, whereas *H3K9* methylation is related to gene repression [46]. However, *H3K27me3* is associated with facultative heterochromatin for gene repression [47]. Heat-induced expression of endogenously reduced repeats can be transmitted for several generations through the trimethylation of histone H3 lysine 9. This was revealed by analysis of the expression profile of Caenorhabditis elegans [48]. In a study performed by Zheng et al. [49]. On a flock of the layer-type L2 strain of Taiwan country chickens (TCCs), it was demonstrated that the abundance of *H3K9me* occurred either as a single posttranslational modification (PTM) or in combination with K14ac, and the abundance of *H3K27me3* occurred either as a single PTM or in combination with K36me, K36me2, or K37me. Decreased *H3K9me* abundance in resistant roosters suggested a negative crosstalk with K14ac, while susceptible roosters showed increased *H3K27me3* abundance, indicating a positive crosstalk with K36me and K37me. As a result, the interaction of these combinatorial PTM may be involved in the adrenal gland’s regulation of the duration and severity of acute heat stress. However, how heat stress affects the ileum and how it affects chickens redox homeostatic genes has not yet been conducted. We hypothesized that supplementing the feed of heat-stressed broilers with Zn would enhance their resilience to heat stress. In this work, we investigated the effects of heat stress on oxidative gene programming in broiler ileum by examining the antioxidant function and the underlying mechanism of *RORγ* and epigenetic modifications.

## 2. Materials and Methods

### 2.1. Experimental Birds, Husbandry, and Diet

This study was performed based on previously published literature [4,50], subsequently. The protocols were slightly modified to be more feasible for this work. A total of 512, 50-day-old Xueshan male broiler chickens were provided by (Jiangsu Lihua Farming Co., Ltd., Changzhou, China). All broilers were acclimated in a room with a controlled temperature of 20 °C to 25 °C for 12 h of light/dark cycles. Broilers had free access to standard water and a diet. After 10 days of adaptation, the broilers were randomly assigned to four groups; 8 replications per group and 16 birds per replicate were used in 42 days. The treatment groups were: (1) no heat stress with basal diet (control or vehicle), (2) heat stress with basal diet (HS), (3) organic zinc with basal diet (Zn), and (4) heat stress with basal diet and organic zinc (HS + Zn). Broilers in the vehicle and Zn were raised in standard conditions from 20 °C to 25 °C throughout the experiment with 55% relative humidity and red fluorescent light with an intensity of about 20 lux. In comparison HS and HS+Zn broilers were subjected to cyclic heat stress using electric heaters at 34 °C to 35 °C for 9:00–17:00, 8 h/d, and the temperature for the remaining periods was set at 28 ± 1 °C with 55% relative humidity. The cages were arranged in a completely randomized manner. The cages were steel cages measuring 90 × 70 × 45 cm in length × width × height. The birds were monitored three times a day to assess their behavior and health conditions. Diets were formulated to meet or exceed the nutrient requirements recommended by the National Research Council (National Research Council 2012) and fed in mash form (Table 1). The broilers diet in the Zn and HS + Zn were supplemented with 60 mg/kg organic zinc, whereas broilers in the vehicle and HS groups were fed a basal diet. The performance, such as body weight and mean daily feed intake, were calculated based on body weight gain and feed intake data recorded every week. At the end of the experiment, 6 birds were randomly selected from each group then the broilers were euthanized, followed by cervical dislocation. After gutting, the segments of the ileal were collected and kept at -80 for further studies. For the ileum histomorphology (n = 4 per treatment), approximately 1 cm of the ileum sample (6 cm proximal to the ileocecal junction) was excised and flushed with 0.9% normal saline to clear the intestinal content before being fixed immediately in a prepared 4% paraformaldehyde solution for intestinal morphological examination.

### 2.2. Antioxidant Indexes

The contents of malondialdehyde (*MDA*), catalase (*CAT*), glutathione peroxidase (*GSH-Px*), and superoxide dismutase (*SOD*) in the ileum were determined by spectrophotometry (PU 8720UV/VIS scanning spectrophotometer) and investigated. Commercial assay kits were provided by the Nanjing Jiancheng Institute of Bioengineering (Nanjing, China), and all procedures were performed following the kit’s protocol.

### 2.3. ROS Levels Assay

Tissues were first homogenized and then washed in PBS, after which the supernatant was collected. Analysis was performed using the OxySelect In Vitro ROS/RNS Assay Kit (Cell Biolabs, STA-347, San Diego, CA, USA). The highly sensitive DCFH is primed for the non-fluorescence assay of DCFH-DiOxyQ. To determine the level of ROS in ileum tissue, the highly fluorescent DCF from the oxidation of DCFH by ROS can be read at 480 nm excitation and 530 nm emissions.

### 2.4. Hepatic Complexes I, III, and V Activities and ATP Content Assay

As previously mentioned, the activities of mitochondrial respiratory chain complexes I, III, and V were measured using appropriate commercial assay kits (Comin Technologies, Co., Ltd., Suzhou, China). The concentration of ileum ATP was measured using an ATP assay kit (Beotime, S0026, Shanghai, China).

### 2.5. Intestinal Morphometry

After 48 h of fixation in paraformaldehyde solutions, the section of small intestinal samples (ileum) was washed, excised, and followed the process of dehydration and rehydration and then embedded in paraffin. The tissues were cut into a thickness of 5 mm and then stained with hematoxylin-eosin. A total of 6 replicates, well-oriented villus-crypt units were selected in triplicate (18 measurements for each sample). Sections were observed under a 10× objective lens, and images were taken using an Olympus microscope (U-TV0.63XC, Tokyo, Japan). Different intestinal morphological parameters such as villus height (VH) and villus width (VW) distance from the tip of the villus to the crypt, crypt depth (CD) distance from the villus base to the submucosa, and the ratio of villus height to crypt depth (VH/CD) were measured using Infinity Analyze software (Version 7, Lumenera Corporation, Ottawa, ON, Canada).

### 2.6. Total RNA Isolation and Real-Time qPCR

Total RNAs were isolated from the ileal tissues (100 mg) using TRIzol reagent (Invitrogen, 15596026, Waltham, MA, USA) following the manufacturer’s instructions. Then, the total RNA concentration was determined using NanoDropOne (Thermo Fisher Scientific, Madison, WI, USA), and the quality was determined using gel electrophoresis. RNAs were reverse transcribed to synthesize complementary DNA (cDNA) using a High-Capacity Reverse Transcription Kit (Applied Biosystems, Foster City, CA, USA). The expressions of the target genes were analyzed using real-time qPCR, as previously described [22]. Specific primers were obtained from NCBI Primer–Blast to perform qPCR using PowerUp SYBR Green Master Mix (Applied Biosystems) on the Quant Studio 3 real-time PCR system (Applied Biosystems, Foster City, CA, USA). The qPCR plate preparation included PCR master mix, consisting of 3 mL cDNA, 5 mL SYBR Green, and 1 mL of primers (forward and reverse, 5 mmol), making the final volume 10 mL. Finally, the target genes were amplified following the standard protocol as previously described [51]. To select the suitable housekeeping gene for the normalization of target genes, tissue samples were also analyzed with three housekeeping genes: glyceraldehyde 3-phosphate dehydrogenase (GAPDH) and beta-actin (β-actin), in triplicate. Beta-actin (β-actin) was the most stable housekeeping gene in the ileum and was selected to normalize the target gene, and the fold change was calculated using the formula 2^−ΔΔCT^.

### 2.7. Western Blotting Analysis

The mixture was used to homogenize ileal tissue after adding phosphatase and protease inhibitors in cell lysis buffer (Biosharp, BL509A Hefei, China). Samples were separated on 10% SDS-PAGE gels after their protein content was equilibrated. After transfer to PVDF membranes (Millipore, IPVH00010, Burlington, CA, USA), samples were blocked for one hour using 5% skim milk. Membranes were first exposed for a 12 h incubation period at 4 °C with some primary antibodies before treatment with HRP-conjugated secondary antibodies. Finally, chemiluminescence was detected using a Dannon 5200 multi-imaging system and a high-sensitivity ECL kit (NCM Biotech, P2300, Suzhou, China).

### 2.8. ChIP-qPCR Measurement

Ileal tissues were cut into small pieces, fixed in 1% formaldehyde for five minutes, and then fixed with ice-cold glycine for five minutes. After that, the samples were resuspended in 50 mM HEPES lysis buffer containing the following components: pH 8.0, 140 mM NaCl, 1 mM EDTA, 10% glycerol, 0.5% NP-40, and 0.25% Triton X-100. After the potatoes were cleaned, samples were suspended in cutting buffer (pH 8.0, 0.1% SDS, 1 mM EDTA, and 10 mM Tris-HCl) and submitted to sonication using a Covaris E220, as directed by the manufacturer. Crude chromatin fragments were incubated with designated antibodies overnight at 4 °C and then treated with protein-G magnetic beads. Chromatin immunoprecipitation was performed as previously described [52,53] with the following modifications. To remove coarse chromatin extracts from ileal tissue, magnetic beads (Thermofisher Scientific, 10004D, Waltham, MA, USA) were first treated with immune serum for 2 h at 4 °C, followed by binding. Then the indicated antibodies were used to incubate the pretreated chromatin solutions overnight at 4 °C. After that, BSA was used to block the protein A beads, and sonicated salmon sperm DNA was added to precipitate the samples. Previous ChIP preparations successfully obtained immunoprecipitated complexes: eluting them with dithiothreitol (20 mM) for 0.5 h at 37 °C, briefly vertexing, and diluting them. The indicated antibodies for secondary ChIP were used to incubate samples overnight at 4 °C, and then qRT-PCR was used to analyze ChIP-ed DNA.

### 2.9. Statistical Analysis

The Shapiro-Wilk test was used to assess the normality distribution of the data. Then, a one-way ANOVA analysis was performed using Tukey’s post hoc tests. GraphPad Prism software 8.0 was used to perform all data analyses. Mean values ± SEM are used to display data. For statistical analysis, a 1-way ANOVA was used. *p* < 0.05 was considered statistically significant.

## 3. Results

### 3.1. Growth Performance

**Figure 1:**
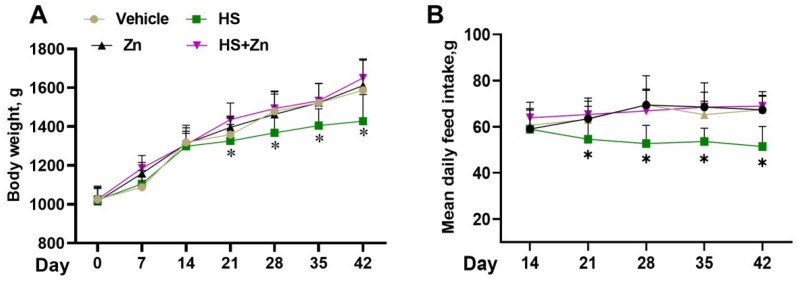
This figure shows the effects of Zn supplementation on the growth performance of the heat-stressed chickens. (**A**) Body weight. (**B**) Average daily feed intake. Data are presented as means ± SD. * *p* < 0.05.

The effects of Zn supplementation on the growth performance of the heat-stressed broiler chickens are shown in Figure 1. There was no significant change (*p* > 0.05) in the body weight of the treatment groups fed with the basal diet until day 14 of the experiment; the body weight was significantly reduced (*p* < 0.05) in heat-stressed chickens compared to the control group on days 21, 28, 35, and 42. However, the inclusion of Zn significantly improved the body weight in the heat-stressed chickens compared to the HS group (Figure 1A). ADFI was significantly decreased (*p* < 0.05) in the heat-stressed chickens from days 21, 28, 35, and 42 compared to the control group, while Zn administration in the heat-stressed chickens (Zn+HS) significantly increased (*p* < 0.05) ADFI from days 21, 28, 35 and 42 compared to the HS group. However, the inclusion of Zn significantly increased the ADFI in the heat-stressed chickens compared to the HS group (Figure 1B).

### 3.2. Intestinal Morphology

**Figure 2:**
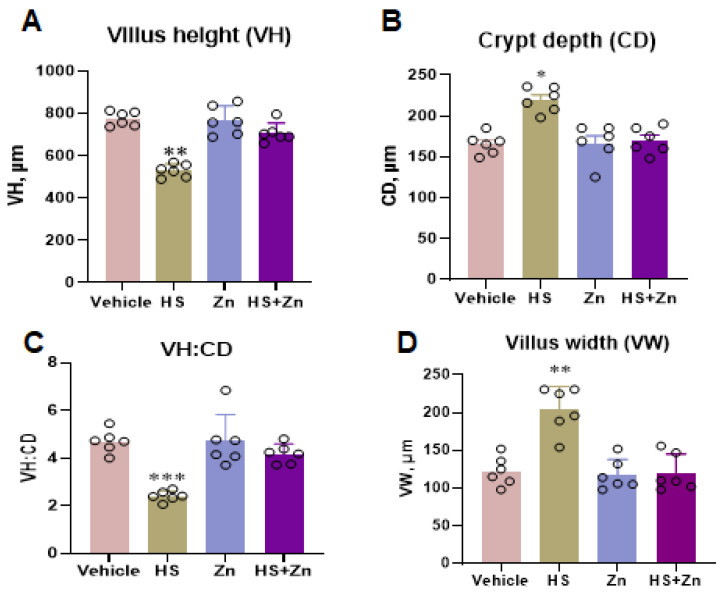
This figure shows the effects of Zn supplementation on the ileum histomorphology of the heat-stressed chickens. (**A**) Villus height (VH). (**B**) Crypt depth (CD). (**C**) Villus height/ Crypt depth (VH/CD). (**D**) Villus width (VW). Data are presented as means ± SD. * *p* < 0.05, ** *p* < 0.01, *** *p* < 0.001, ° = the number of replication.

As shown in Figure 2, the VH and VH: CD were significantly reduced (*p* < 0.05) in the HS group compared to the control group, while the administration of the Zn in heat-stressed chickens significantly increased (*p* < 0.05) the VH and VH: CD compared to the HS group (Figure 2A,C). Moreover, the results showed that CD and VW of the ileal were significantly increased (*p* < 0.05) in the HS group compared to the control group, while supplementing of the Zn exhibited the effects of these parameters compared to the HS group (Figure 2B,D).

### 3.3. Oxidative Stress in the Ileal of Broiler Chickens

**Figure 3:**
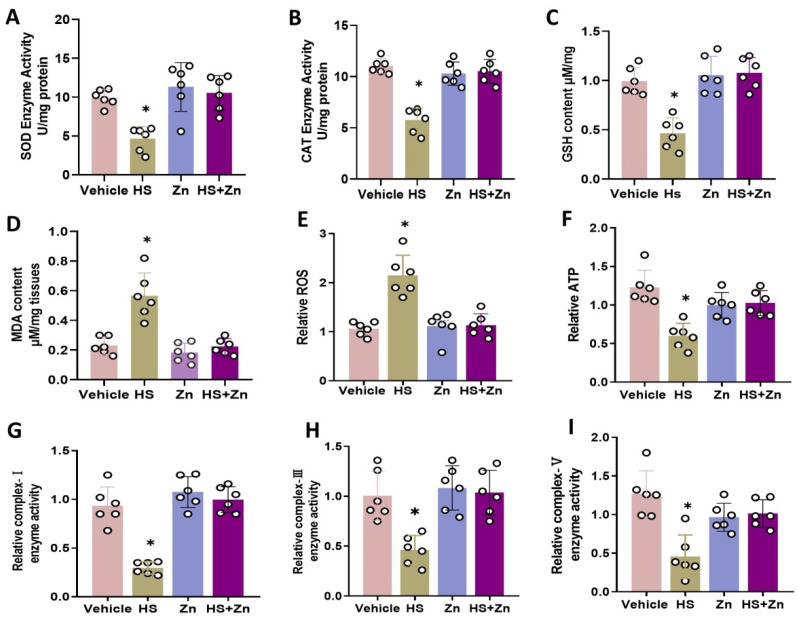
Effects of Zn supplementation on the oxidative stress of the heat-stressed chickens. (**A**) Superoxide dismutase (*SOD*). (**B**) Catalase (*CAT*). (**C**) Glutathione peroxidase (*GSH*). (**D**) Malondialdehyde (*MDA*). (**E**) Reactive oxygen species (*ROS*). (**F**–**I**) The relative parameters of mitochondria *ATP*, enzyme complex-I, complex-III, and complex-V. Data are presented as means ± SD. * *p* < 0.05, ° = the number of replication.

As shown in Figure 3, the ileal *SOD*, *CAT*, and *GSH* activities decreased (Figure 3A–C), while ileal *MDA* levels significantly (*p* < 0.05) increased in the heat-stressed chickens compared to the control group (Figure 3D). Moreover, *ROS* production was significantly increased (*p* < 0.05) in the heat-stressed chickens compared to the control group (Figure 3E). Heat-stressed chickens had significantly reduced the content of ATP and enzyme activities of mitochondrial complexes I, III, and V compared to the control group (Figure 3F–I). Administration of organic Zn significantly (*p* < 0.05) restored these processes compared to the HS group.

### 3.4. Ileal Gene Expression

**Figure 4:**
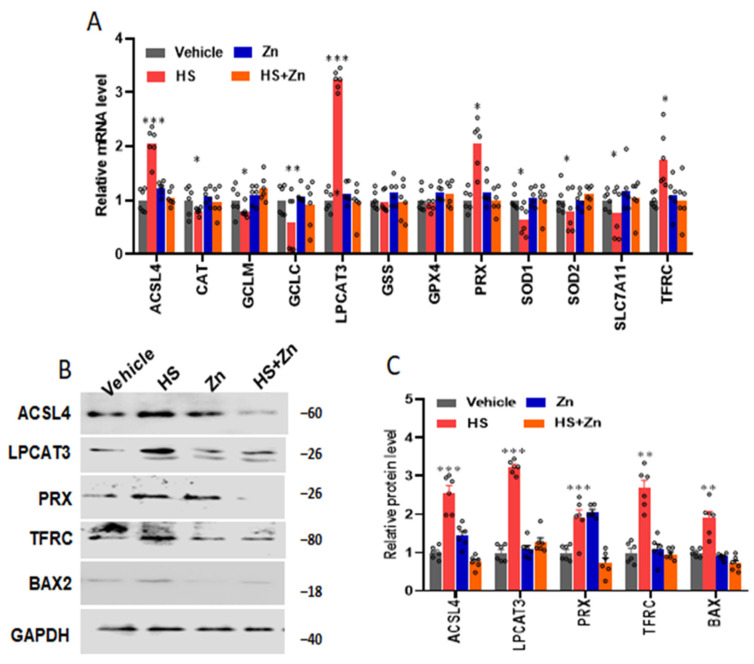
Effects of Zn supplementation on the ileum expressions of genes included in antioxidation of the heat-stressed chickens. (**A**) mRNA expression changes of the antioxidants related. (**B**,**C**) protein levels changes of the antioxidants related. Data are presented as means ± SD. * *p* < 0.05, ** *p* < 0.01, *** *p* < 0.001, ° = the number of replication.

As displayed in Figure 4, the mRNA expression of *CAT*, *GCLM, GCLC*, *SOD1*, *SOD2,* and *SLC7A11* significantly decreased, and the mRNA expression of ACSL4, LACLT3, PRX, and *TFRC* significantly increased in the heat-stressed chickens compared to the control group (Figure 4A). However, they were significantly restored (*p* < 0.001) in the Zn-supplemented groups compared to the HS group. The protein levels of *ACSL4*, *LPCAT3*, *PRX*, *TFRC*, and *BAX2* significantly increased in the heat-stressed chickens compared to the control, while Zn supplementation significantly reduced (*p* < 0.05) the protein levels of *ACSL4*, *PRX*, *TRFC*, and *BAX2* in the heat-stressed chickens compared to the HS (Figure 4C).

### 3.5. Histone Modifications Facilitate the Transcriptional Suppression of Antioxidants

**Figure 5:**
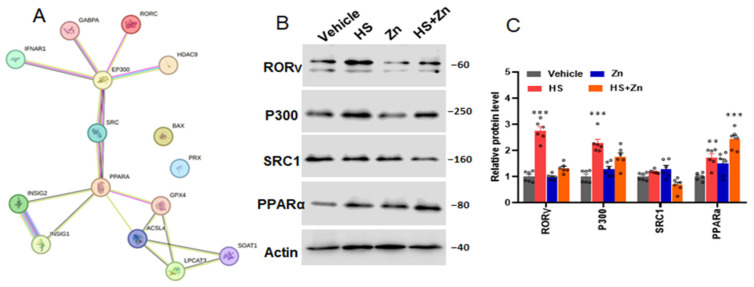
Effects of Zn supplementation on the ileum transcriptional activation of gene expression of the heat-stressed chickens. (**A**) The interactions among *RORγ*, *SRC*, *PPARα*, and core proteins involved in cholesterol metabolism during transcriptional regulation were predicted by Search Tool for the Retrieval of Interacting Genes (STRING). (**B**,**C**) Western blotting analysis was performed to evaluate the expression of nuclear *RORγ*, *P300*, *SRC*, *PPARα*, at the protein level. Data are presented as means ± SD. ** *p* < 0.01, *** *p* < 0.001, ° = the number of replication.

As shown in Figure 5, the levels of *RORγ* and P300 significantly (*p* < 0.001) increased in the heat-stressed chickens compared to the control group. The protein levels of *SRC1* were not affected by heat stress compared to the control; however, they were significantly downregulated (*p* < 0.001) in the Zn-supplemented groups compared to the HS group. Additionally, the protein levels of PPARα significantly improved in HS compared to the control groups, thus it was significantly upregulated (*p* < 0.001) in the HS + Zn group compared to the control and Zn groups (Figure 5C).

**Figure 6:**
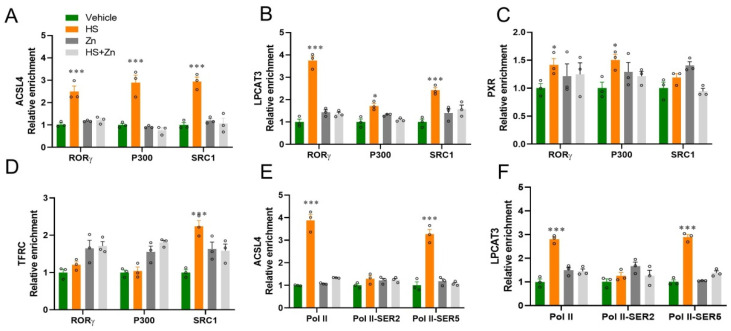
Effects of Zn supplementation on the ileum, the enrichment of *RORγ, P300*, and *SRC1* at target loci of *ACSL4*, *LPCAT3*, *PXR*, and *TFRC* genes and their physical interaction of the heat-stressed chickens. (**A–D**) ChIP-qPCR analyses of *RORγ*, *P300*, and *SRC1* occupancies at the locus of *ACSL4, LPCAT3, PXR*, and *TFRC*. (**E**,**F**) The relative enrichment of coactivator RNA polymerase II, RNA polymerase II-SER2, and RNA polymerase II-SER5 at the locus of *ACSL4* and *LPCAT3*. Data are presented as means ± SD. * *p *< 0.05, *** *p* < 0.001, ° = the number of replication.

**Figure 7:**
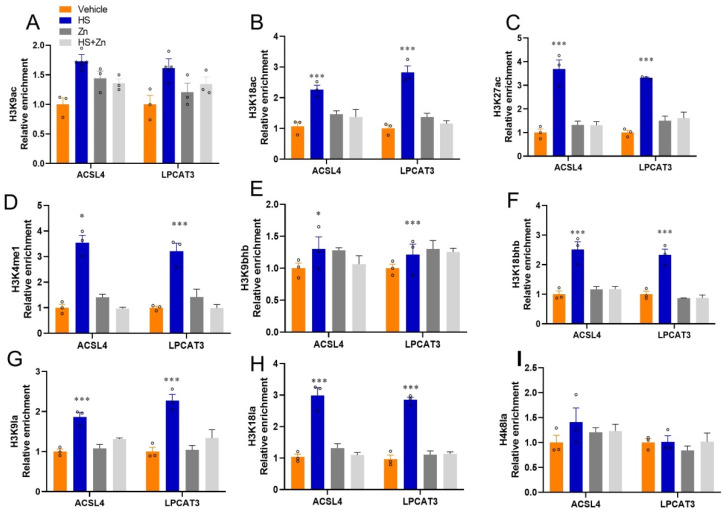
The effects of Zn supplementation on the ileum, histone modification at the locus of *ACSL4* and *LPCAT3* in heat-stressed chickens. (**A**–**I**) The relative enrichment of histone marks’ (*H3K9ac*, *H3K118ac*, *H3K27ac*, *H3K4me1*, *H3K9bhb*, *H3K18bhb*, *H3K9la*, *H3K18la*, and *H4K8la*) occupancy was analyzed by ChIP-qPCR. Data are presented as means ± SD. * *p* < 0.05, *** *p* < 0.001, ° = the number of replication.

The enrichment of *RORγ*, *P300*, and *SRC1* at the target loci of antioxidant genes has been shown in (Figure 6). The enrichment of *RORγ*, P300, and *SRC1* at target loci of *ACSL4*, *LPCAT3*, and *PRX* significantly upregulated (*p* < 0.001) in the heat-stressed compared to the control group (Figure 6A–C). In addition, the enrichment of Pol II and Pol II-SER5 at target loci of *ACSL4* and *LPCAT3* significantly upregulated (*p* < 0.001) in the heat stress compared to the control group; however, they were significantly downregulated (*p* < 0.05) in the Zn-supplemented groups compared to the HS group. While Pol II-SER2 has no significant change at target loci of *ACSL4* and *LPCAT3* between the groups (Figure 6E,F). We then performed ChIP-qPCR to detect the transcriptional activation-linked histone marks *H3K9ac*, *H3K18ac*, *H3K27ac*, *H3K4me1*, *H3K9bhb*, *H3K18bhb*, *H3K9la*, *H3K18la* and *H3K8la* at the locus of *ACSL4* and *LPCAT3* in ileal (Figure 7). The histone marks *H3K9ac*, *H3K18ac*, *H3K27ac*, *H3K4me1*, H3K9bhb, H3K18bhb, and related to transcriptional activation were significantly (*p* < 0.001) upregulated by heat-stress at the developers of *ACSL4* and *LPCAT3* respectively, (Figure 7A–F). Moreover, enrichment of *H3K9la* and *H3K18la* at target loci of *ACSL4* and *LPCAT3* significantly upregulated (*p* < 0.05) in the heat-stressed compared to the control group (Figure 7G,H), while they were significantly downregulated (*p* < 0.05) in the Zn-supplemented groups compared to the HS group. However, *H4K8la* has not been affected yet (Figure 7I).

## 4. Discussion

Broiler chickens are the best source of animal-based protein for human consumption. However, high temperatures, oxidative stress, and detrimental impacts on the well-being and productivity of broilers due to adverse physiological characteristics result in substantial financial losses for the industry [9,17,54]. In poultry, feed is reduced by 5% for every 1 °C rise in the temperature range of 32-38 °C [9]. Thus, it is necessary to use sustainable methods to reduce heat stress in broilers. In the present study, we found that heat stress decreased body weight and ADFI in broilers. Our finding was in agreement with Abuajamieh et al. [55] and Xiao et al. [50] who reported that HS decreased broiler performance, as manifested by the decrease in ADFI and body weight. In hot weather, birds need to breathe in order to relieve the heat. According to a previous study, birds cannot breathe and feed at the same time while under heat stress. For this reason, when birds are exposed to high temperatures, they pant more than they eat [56]. Another explanation is that birds that are hyperthermic attempt to reduce their metabolic heat by eating less [57]. Moreover, we revealed that dietary Zn supplementation increased body weight and ADFI in HS broilers. This finding was in line with Hu Ping et al. [4], who observed that, in contrast to the control group fed the standard diet, broilers supplemented with zinc had a much higher feed-to-weight ratio during the experimental period under heat stress. Further, this study revealed that the dietary supplementation of Zn improved the redox system, intestinal health, and heat-induced oxidative stress broilers. This positive outcome of the study suggests that Zn could be a beneficial supplement in broilers for combating the negative effects of heat stress.

The intestinal mucosal structure, made up of finger-like projections called villi, is important for nutrient absorption in addition to basic health indicators. Microvilli, microscopic structures, form additional villi. The surface area of the small intestine is increased by villi, which are essential for the absorption of nutrients [4]. Heat stress is one of many variables that can affect gut health by altering villi length and reducing the absorptive capacity of the small intestine [58]. Previous studies have shown that heat stress in broiler chickens can damage villi at the apex of the small intestine, significantly reducing the height of intestinal villi [59,60,61]. In agreement with previous studies, our finding revealed that broiler chicken under heat stress exhibited a decrease in VH and VH: CD and increased CD and VW in ileum, while the zinc supplementation restored these. Therefore, supplementing with zinc may help to enhance and ameliorate intestinal morphology.

HS induces oxidative stress, which is mediated by the production of reactive oxygen species (*ROS*) [3], with antioxidant enzymes acting as an important defense mechanism to protect tissues from *ROS* [62]. In general, oxidative stress is a physiological stress caused by an imbalance between the ability of the antioxidant system to scavenge *ROS* and the formation of *ROS* as a result of metal exposure. This imbalance results in damage to macromolecules, changes in the redox state of cells, and the regulation of gene expression [63]. *SOD* activity converts O_2_^−^ and H^+^ into less reactive H_2_O_2_, which is the first line of defense against oxidative stress in chickens [64]. Most animals are protected from oxidative stress by *CAT* and *GPH* activity, which convert H_2_O_2_ to water and oxygen; *CAT* also regulates cellular *ROS* production, which is involved in the regulation of cellular signaling [65]. Also, *CAT* is important in keeping low H_2_O_2_ levels in the normal range, which helps maintain cellular homeostasis and adapt to stress. In the present study, we investigated the expression of several antioxidant-related genes in the ileum to understand the mechanism by which Zn supplementation improves the redox system. The activity of *CAT*, *SOD*, *GSH,* and *ATP* decreased in heat-stressed broiler chickens, while the supplementation of Zn increased their activity in heat-stressed broilers. Increased concentrations of *MDA* and *ROS* in the heat-stressed treatment indicate the acclimatization of broilers to chronic heat stress. However, heat stress leads to excessive generation of free radicals and alters redox dynamics, causing oxidative damage to proteins, lipids, and nucleic acids. Free radicals, *ROS*, and *RNS*, are normally maintained at physiological levels [4]. Lipid peroxidation produces *MDA*, which is a by-product that puts cells under toxic stress and is used as a biomarker for measuring an organism’s amount of oxidative stress. Consequently, antioxidant activity is typically associated with a living system defense mechanism [9]. Therefore, heat stress damages gut health by inducing oxidative stress, compromising gut health and nutrient absorption, which was improved by Zn supplementation. Energy homeostasis, apoptosis, metabolic signaling, intracellular calcium balance, and lipid synthesis depend on mitochondria, double-membrane organelles that catalyze oxidative phosphorylation to adenosine triphosphate (*ATP*) [66]. The process of oxidative phosphorylation in mitochondria complexes I, III, and IV uses most of the energy in cells and is also a contributor to the cellular production of HS. Under normal physiological conditions, HS production is safely controlled by antioxidant defence mechanisms and is ultimately not harmful [67]. In the present study, mitochondria complexes I, III, and IV increased in heat-stressed broilers supplemented with Zn compared to the HS group. It has been consistently proposed that some complex I and III enzyme activities support mitochondrial respiration in response to HS.

The redox-sensitive nuclear transcription factor *Nrf2* is translocated to the nucleus in response to heat stress-induced oxidative stress in the broiler. There, it binds to the promoter region of the antioxidant response element in DNA, resulting in the production of various antioxidants [68]. Therefore, various antioxidant-related genes, including *ACSL4, CAT, GCLM, GCLC, LPCAT3, GSS, GPX4, PRX, SOD1, SOD2, SLC7A11*, and *TFRC*, were examined to develop a better understanding of the antioxidant status of heat-stressed birds. The cell transmembrane protein *SLC7A11* is part of the light chain of the xc-system, which brings extracellular cysteine into cells for *GSH* formation and cysteine synthesis. *SLC7A11* is an essential gateway for redox homeostasis by maintaining cellular levels of *GSH* that counteract cellular oxidative stress and reduce ferroptosis [69]. In the process of ferroptosis, *GPX4* acts as a master regulator; its unique role is to stop lipid peroxidation by converting lipid hydroperoxides into non-toxic lipid alcohols [70]. *GPX4* activity decreases, leading to an intracellular peroxide accumulation that exacerbates ferroptosis [71]. By activating *SLC7A11* and *GPX4*, it may prevent ferroptosis. According to Kwata and Hara [72], *ACSL4* is an important enzyme associated with lipid metabolism in vivo, primarily facilitating the synthesis of fatty acids with a 12-20 carbon chain length. Superoxide dismutase (*SOD*) occurs in two isoforms, *SOD1* and *SOD2. SOD2* is composed of manganese (Mn)- containing enzymes in mitochondria, *SOD1* or cytosolic Cu/ZnSOD, mostly found in the cytoplasm, nucleus, mitochondrial intermembrane spaces, lysosomes, and peroxisomes [73]. The most common type of free radical generated within cells is superoxide radicals [74]. Because *SODs* catalyze the conversion of the superoxide radical to hydrogen peroxide, they are considered primary components of the cell’s initial line of defense against antioxidants [5]. In the terrestrial cycle, *LPCAT3* is an important membrane acyltransferase that produces C20:4 phospholipids. It is linked to several important biological processes, including intestinal fat absorption, lipoprotein assembly, and ferroptosis [75]. A cell surface receptor required for cellular iron uptake is encoded by the transferrin receptor (*TFRC*) gene. Receptor-mediated endocytosis is the movement of iron from the outside to the inside of the cell, necessary for cell growth [76]. In the present study, *CAT, GCLC, SOD1, SOD2*, and *SLC7A11* were decreased and *ACSC4, LPCAT3, PRX,* and *TFRC* were increased in the heat-stressed chickens compared to the control group, while the zinc supplementation restored these processes. Previous research has demonstrated the downregulation of *SLC7A11, GCLC,* and *GCLM* due to mRNA-mediated post-transcriptional regulation [51]. According to that research, microminerals found in zinc may have a similar effect, upregulating antioxidant genes and reducing intracellular reactive oxygen species (*ROS*) and lipid peroxidation in heat-stressed chickens. Further studies are needed to investigate the effect of heat-stressed birds supplemented with Zn on antioxidant enzymes in broilers.

The nuclear receptor transcription factor *ROR* gamma, also known as *RORγ*, is important for the development and function of the immune system, particularly for Th17 cell differentiation and the control of inflammation. *RORγ* has highlighted the crucial role of the ileum in nutrient absorption and intestinal immunity, emphasizing its function within this section of the small intestine in chickens [77]. *RORγ* binds directly to MVK pathway genes and induces transcription [78]. As a transcription coactivator, *P300* was identified; it acetylates core histones, promotes chromatin decondensation, and facilitates the development of the basal transcription machinery [79]. Acetylation is thought to play a role in the Nrf2-dependent oxidative response, with the recent finding that P300 acetylates *Nrf2* and increases promoter-specific DNA binding during oxidation [80]. In this study, it was found that heat-stressed broilers increased the protein levels of *RORγ* and *P300* while decreasing in heat-stressed broilers supplemented with Zn compared to the HS group, but did not affect *SRC1*. Steroid receptor coactivator 1 (*SRC-1*), helps DNA binding by adding basic amino acid residues to each helix. Furthermore, most *SRCs* contain a region that binds *CBP* to interact with *p300/CBP*. Consequently, these *SRCs* are often located in the *p300/CBP* epigenetic regulatory complex [81]. In contrast, *SRC1* in the central nervous system is primarily involved in neuron plasticity, neural stem cell differentiation, and motor learning [82]. The transcription coactivator *SRC1* is involved in energy expenditure in adipose tissue and liver and intestine [83]. *P300* (E1A binding protein) and CBP (CREB binding protein), which are paralogous proteins, are essential for the transcriptional regulation of gene expression [84]. The potential of P300-specific inhibitors has been shown by recent investigations into their therapeutic applications in autoimmune disorders and cancer [85]. Specifically, inhibitors directed against the *P300* bromodomain affect Treg cell growth and function, providing a method to enhance effector responses to *ROS* [86]. By interacting with *PPAR* response components in gene promoter regions, the *PPAR* isoform *PPAR-α* regulates the expression of genes involved in inflammatory responses, glucose and lipid metabolism, and other functions [52,87]. According to Sun et al. [88], *PPAR-α* is a key regulator of energy metabolism, mitochondrial function, and peroxide isoenzyme activity. Endogenous ligands such as palmitic acid, stearic acid, oleic acid, arachidonic acid, eicosapentaenoic acid, and fibrates (clofibrate, gemfibrozil, nafenopin, bezafibrate, and fenofibrate) and fibrates reduce or inhibit angiogenesis, lipotoxicity, and oxidative stress [89]. In this study, supplementing Zn increased protein levels for *PPARα* in heat-stressed broilers. It has been demonstrated that *PPAR-α* regulates the expression of genes associated with fatty acid oxidation and is a key regulator of energy balance. It can also control the development of autophagy [90]. We provide evidence that heat-stressed chickens increased *RORγ, SRC1*, and *P300* enrichments at the key genes *ACSL4, LPCAT3, PXR*, and *TFRC*. As transcriptional binding and initiation are the primary mechanisms governing gene regulation, the up-regulated expression of *ACSL4, LPCAT3*, *PXR*, and *TFRC* genes may be responsible for the increasing binding enrichment of the transcriptional regulators *RORγ, SRC1*, and *P300.* However, Zn supplementation down-regulated the expression of ACSL4, *LPCAT3, PXR*, and *TFRC* genes, and may be responsible for the decreased binding enrichment of the transcriptional regulators *RORγ, SRC1*, and *P300* in heat-stressed broilers. Moreover, in the present study, heat-stressed chickens increased *ACSL4* and *LPCAT3* enrichments at the key genes Pol II, Pol II-SER2, and Pol II-SER5, whereas supplementing Zn decreased the process in heat-stressed broilers.

Active transcription of genes is often associated with acetyl and non-acetyl histone acylations. The fact that multiple histone acetyl/acyl-transferases are often organized into substantial complexes containing histone acylation reader modules suggests that the generation of histone acetyl/acylation states is probably hierarchical [91]. The Lysine 18 residue of histone H3 is post-translationally modified to add a lactyl group, known as *H3K18la* or *H3K18ac*. Histone 3 lysine 27 (*H3K27ac*) and Histone 3 lysine 9 (*H3K9ac*), linked to active promoter and enhancer regions, are acetylated by *P300/CBP*, facilitating transcription through epigenetic mechanisms [86]. Referring to the broad domain of *H3K27ac* as an enhancer or super-enhancer is a clear overstatement, as it ignores the complex relationships that control gene regulation [84]. Histone H3 trimethylated at lysine 4 (*H3K4me1*) coordinates multiple signaling cascades such as RNA splicing, elongation, and transcription development [92]. Different from other broad epigenetic traits such as super-enhancers, broad *H3K4me1* is linked to high transcription elongation and enhancer activity, resulting in unusually high gene expression [93]. Numerous unique histone PTMs are present in nucleosomes of the broad *H3K4me1* domain [94]. Like other PTMs, Kbhb was first identified in histones [95]. Residues that can be acetylated are found at many histone Kbhb sites. Histone 3 lysine 9 (*H3K9bhb*) and Histone 3 lysine 18 (*H3K18bhb*) is one of the most researched Kbhb residues because it is acetylated (*H3K9ac*) in promoter regions [96] due to its association with active gene expression. *H3K9bhb* chromatin immunoprecipitation (ChIP) experiments have been used in several studies to detect BHB-regulated genes under starvation, BHB treatment, or ketogenic diets [96,97]. Furthermore, histone *H3K9bhb* is linked to starvation-responsive gene regulation and dissociates some of these genes from genes marked by *H3K9ac* and *H3K4me3*, suggesting a specialized function in coupling starvation-responsive metabolic and epigenetic regulation [98]. H3K9me1, *H3K18ac*, and *H3K9bhb* epigenetically affected *ACSL4* and *LPCAT3*, and they also reduced *ACSL4* and *LPCAT3* expression, resulting in an increase in lipid peroxidation [99]. Studies indicate that this modification is important for tissue-specific enhancer activity, gene regulation, and physiological processes [100]. Furthermore, we also found that the *H3K4me1, H3K9ac, H3K27ac, H3K9bhb, H3K18bhb* and *H3K18ac* enrichments on *ACSL4* and *LPCAT3,* and *H3K9la, H3K18la* and *H4K8la* enrichments on *ACSL4* and *LPCAT3* were consistently diminished in the ileum. Elevated *ACSL4* and *LPCAT3* activities may indicate an adaptive response due to elevated oxidative stress [101].

## 5. Conclusions

In conclusion, the results of this study reveal that organic zinc supplementation has a beneficial effect on the intestinal histology and regulation of heat stress index genes in broiler chickens. Zinc administration resulted in an increase in VH and VH:CD, and the reduced CD and VW of the ileum, as well as a decrease in the enzymatic activities of *ROS* and *MDA*. The expression of key genes involved in oxidative stress was downregulated, and the regulation of these genes was mediated through nuclear receptor *RORγ* and histone modifications. These results suggest that organic zinc supplementation may be a potential strategy for improving the resilience of broiler chickens to heat stress.

## Figures and Tables

**Table 1:** Composition and nutrient levels of the basal diets for 60–102 broilers (as-fed basis).

Item	Basal Diet
Ingredient (%)	
Corn	76.27
Soybean meal	19.50
Soybean oil	1.38
DL-Met	0.12
L-Lys	0.13
CaHPO_4_·2H_2_O	0.79
CaCO_3_	1.15
NaCl	0.30
Micronutrients ^1^	0.26
Cornstarch + zinc	0.10
Nutrient levels composition	
ME, Kcal/kg	3037
Crude protien %	15.31
Lys, %	0.81
Met, %	0.36
L-Thr, %	0.57
Try, %	0.16
Met+Cys, %	0.60
Ca, %	0.69
P, %	0.45
Nonphytate P, %	0.22
Zinc mg/kg	18.33

^1^ VA 6000 IU, VD3 2250 IU, VE 16.5 IU, VK3 1.5 mg, VB1 1.5 mg, VB2 4.8 mg. VB6 2.25 mg, VB12 0.015 mg. Pantothenic acid calcium 7.5 mg, Niacin 27 mg, Folic acid 0.75 mg, Biotin 0.075 mg, Choline 750 mg, Cu (CuSO_4_·5H_2_O) 7 mg, Fe (FeSO_4_·7H_2_O) 40 mg, Zn (ZnSO_4_·7H_2_O) 0 mg, Mn (MnSO_4_ H_2_O) 40 mg, Se (Na_2_SeO_3_) 0.15 mg, and I (Ca(IO_3_)2·H_2_O) 0.5 mg.

## Data Availability

The data presented in this study are available in the corresponding author.
